# Structure modification of an antibiotic: by engineering the fusaricidin bio-synthetase A in *Paenibacillus polymyxa*

**DOI:** 10.3389/fmicb.2023.1239958

**Published:** 2023-09-26

**Authors:** Yunlong Li, Sanfeng Chen

**Affiliations:** ^1^Chengdu NewSun Crop Science Co. Ltd., Chengdu, China; ^2^State Key Laboratory of Agrobiotechnology, College of Biological Sciences, China Agricultural University, Beijing, China

**Keywords:** gene engineering, [ΔAla^6^] fusaricidin LI-F07a, structure modification, homologous recombination, biocontrol agent

## Abstract

Fusaricidin, a lipopeptide antibiotic, is specifically produced by *Paenibacillus polymyxa* strains, which could strongly inhibit *Fusarium species* fungi. Fusaricidin bio-synthetase A (FusA) is composed of six modules and is essential for synthesizing the peptide moiety of fusaricidin. In this study, we confirmed the FusA of *Paenibacillus polymyxa* strain WLY78 involved in producing Fusaricidin LI-F07a. We constructed six engineered strains by deletion of each module within FusA from the genome of strain WLY78. One of the engineered strains is able to produce a novel compound that exhibits better antifungal activity than that of fusaricidin LI-F07a. This new compound, known as fusaricidin [ΔAla^6^] LI-F07a, has a molecular weight of 858. Our findings reveal that it exhibits a remarkable 1-fold increase in antifungal activity compared to previous fusaricidin, and the fermentation yield reaches ~55 mg/L. This research holds promising implications for plant protection against infections caused by *Fusarium* and *Botrytis* pathogen infection.

## 1. Introduction

Non-ribosomal peptide synthetase (NRPS) is a microbial systemic enzyme that produces secondary metabolites such as lipopeptide antibiotics (Shokrollahi et al., 2021). In general, NRPS enzymes consist of multiple modules, with each module capable of accommodating several domains, including the condensation domain, adenylation (A) domain, thiolation (T) domain, epimerization (E) domain, and thioester (TE) domain (Marahiel, 2016; Izoré et al., 2021). In certain literature, the T domain is alternatively referred to as the peptidyl carrier protein (PCP) domain (Rüschenbaum et al., 2022). Additionally, it has been demonstrated that the C domains contain two catalytic tunnels that connect the donor-PCP and acceptor-PCP domain-binding sites to the active site. These tunnels serve as the pathway for the donor and acceptor substrates to access the active site (Samel et al., 2007).

The structure of a three-module NRPS contains an initiation module (core A-T domain), an elongation module (core C-A-T domain) that can be followed by an E domain, and a termination module (core C-A-T-TE domain) (Calcott and Ackerley, 2015; Izoré et al., 2021). The bio-synthesis process of peptide moiety can be divided by these domains into several steps, beginning with post-translational modification of each T domain by attachment of a 4′-phosphopantetheine (4′-pp) cofactor. The 4′-pp group serves as a flexible arm that facilitates the coordination of substrate movement between catalytic sites (Kittilä et al., 2016; Zhang et al., 2022). Within each module, the A domain plays a crucial role in recognizing a specific monomer and activating it as an aminoacyl adenylate. Subsequently, it attaches the activated monomer to the 4′-pp group located on the adjacent T domain (Schwarzer et al., 2002; Zhu et al., 2019). In a sequential fashion, beginning with the first module, each C domain facilitates the formation of peptide bonds by linking the donor substrate, which is attached to the upstream T domain, with the acceptor substrate, which is attached to the downstream T domain (Wheadon and Townsend, 2021). Before the condensation reaction takes place, it is possible that each T domain may require interaction with additional tailoring domains, such as the E domain. The E domain plays a role in modifying the substrate carried by each module, including the ability to catalyze racemization (Kim et al., 2022). Once the peptide chain reaches the termination module, the product is released by a thioesterase (TE) domain through hydrolysis or intramolecular cyclization. Following this, the synthesis cycle can be repeated to generate multiple copies of the same peptide (Marahiel, 2016; Hühner et al., 2019).

Fusaricidin is an important antibiotic family produced by *Paenibacillus polymyxa* with great potential use in medical and agricultural applications (Kajimura and Kaneda, 1996; Lee et al., 2012; Haron et al., 2019; Jeong et al., 2019). So far, 14 fusaricidin members have been reported, consisting of an invariable 15-guanidino-3-hydroxypentadecanoic acid and a variable cyclic hexapeptide (Deng et al., 2011; Vater et al., 2015; Reimann et al., 2017). The variable cyclic hexadepsipeptide moiety of fusaricidin usually contains six amino acid residues: L-Thr^1^, X^2^, X^3^, D-allo-Thr^4^, X^5^, and D-Ala^6^ (Han et al., 2012). Among the family, fusaricidin LI-F07a has better antimicrobial activity than other fusaricidin analogs. Based on the current knowledge, L-Thr^1^, D-Val^2^, L-Phe^3^, D-allo-Thr^4^, D-Asn^5^, and D-Ala^6^ constitute the cyclic hexadepsipeptide moiety within LI-F07a (Reimann et al., 2017).

The discovery of penicillin in 1928 started the golden age of natural product antibiotic discovery that peaked in the mid-1950s (Hutchings et al., 2019). Since then, a gradual decline in antibiotic discovery and development and the evolution of antibiotic resistance in many human pathogens has led to the current antimicrobial resistance crisis (Larsson and Flach, 2022). We believe that the future of antibiotic findings looks bright as new technologies such as genome mining and editing are deployed to discover new antibiotics with diverse bioactivities (Albarano et al., 2020).

In our previous studies, we reported the strain WLY78 produces fusaricidin, and the fermented strain could be used for bio-controlling the *Fusarium* head blight in wheat (Li and Chen, 2019; Li et al., 2021). While acknowledging the effectiveness of this biocontrol strain, we are not content with maintaining the present status. Our primary motivation is to proactively prevent the potential emergence of microbial resistance issues, which serves as a driving force for our continuous advancements. Our objective is to optimize the NRPS enzymes involved in the synthesis of existing antibiotics strategically. This optimization aims to enhance the effectiveness of antibiotics. In our current study, we have created a new fusaricidin antibiotic by modifying the FusA. Briefly, we analyzed the function of FusA and confirmed it could biosynthesizes fusaricidin LI-F07a. Then, each module within FusA was reorganized by gene modification. Moreover, we found that one of the engineered strains produced a novel fusaricidin derivative. The structure of this novel compound was identified as [ΔAla^6^] fusaricidin LI-F07a. Meaningfully, this new compound exhibits stronger activity than fusaricidin LI-F07a in inhibiting the pathogen fungi *F. asiaticum*. Finally, this novel substance was successfully applied to prevent plants from being infected by pathogens: *Fusarium oxysporum* and *Botrytis cinerea*.

## 2. Materials and methods

### 2.1. Microorganisms, plasmids, and culture conditions

The source of strains and plasmids is summarized in Supplementary Table S1. *Escherichia coli* DH5α was cultivated at 37°C in Luria–Bertani (LB) broth for cloning plasmids. *P. polymyxa* strain was cultivated at 30°C in Katznelson–Lochhead (KL) broth for the production of fusaricidin and its derivatives (Paulus and Gray, 1964). The pathogens *Fusarium asiaticum*, *Fusarium oxysporum*, and *Botrytis cinerea* were cultivated at 28°C in a potato dextrose agar (PDA) medium. A temperature-sensitive shuttle vector, pRN5101, containing the ori(Ts) and erm^R^ of pE194ts and the oriEc, amp^R^, and multicloning region of pBR322, was used for gene deletion in *P. polymyxa* (Villafane et al., 1987; Lereclus et al., 1992). If necessary, LB broth was solidified using 1.5% agar, and the antibiotics were added at the following concentrations: 100 μg/mL ampicillin (95% purity, for selecting *E. coli* transformants) and 5 μg/mL erythromycin (95% purity, for selecting *P. polymyxa* transformants).

### 2.2. Bioinformatics analysis of FusA for its substrate prediction

The genome of our strain WLY78 is genome sequenced (please refer to GenBank: ALJV00000000). The *fusA* gene and the *fus* gene cluster sequence have been deposited to GenBank (AYC81015.1 and MH368541.1). For the A domains alignment, the amino acid sequence of each A domain was extracted from FusA via the antiSMASH bacterial version program (Blin et al., 2023). The GenBank numbers of these A domains from different NRPSs are listed in Supplementary Table S2. The “ten code residues” were acquired from a previous study (Stachelhaus et al., 1999). Ten code residues located at positions 235, 236, 239, 278, 299, 301, 322, 330, 331, and 517 within each A domains were extracted and subsequently combined to form a new amino acid sequence for the purpose of sequence alignment. The phylogenetic tree was constructed to predict the substrate specificity by using the software MEGA 5.0.

### 2.3. Modification of FusA

The cyclic peptide moiety of fusaricidin is composed of six amino acids that were assembled by six modules. To produce a novel fusaricidin derivative, each of the six modules (M1, M2, M3, M4, M5, and M6) within FusA was deleted *via* homologous recombination (Supplementary Figure S1).

The method of assembling fragments and plasmids was carried out according to the manufacturer’s instruction (Gibson Assembly Kit, ThermoFisher). Briefly, two homologous arms (each with 1 kb in length) flanking the deleting module were PCR amplified from the genomic DNA of *P. polymyxa*. The two homologous arms were assembled into the suicide plasmid pRN5101 digested by *Bam*HΙ (0.75 U/μL, TaKaRa), yielding six recombinant plasmids: pRN-M1, pRN-M2, pRN-M3, pRN-M4, pRN-M5, and pRN-M6. Each of these recombinant plasmids was transformed in *P. polymyxa*, and the single-crossover transformants were selected for erythromycin resistance as previously described (Zhang et al., 2013). Subsequently, the double-crossover (marker-free deletion) mutants were selected from the initial erythromycin resistance transformants after several rounds of non-selective growth at 39°C and confirmed by PCR amplification. Finally, we acquired six mutant strains that carry the engineered FusA: ΔM1, ΔM2, ΔM3, ΔM4, ΔM5, and ΔM6. The primers for the PCR are listed in Supplementary Table S3.

### 2.4. Antifungal activity assays

To assess the antifungal activity of wild-type strain and its genetically engineered strains, the inhibition zone against the fungus *F. asiaticum* was measured as previously described (Li and Chen, 2019). In brief, *F. asiaticum* was inoculated at the center of the PDA medium. Next, 1 μL of strain cell suspensions (10^7^ CFU/mL) was inoculated around the fungus, maintaining a constant distance of 2 cm. All the plates were then incubated at 28°C for 4 days, and the inhibition effect on fungi hyphae growth was recorded.

To test the antifungal activity of purified component, including LI-F07a and [ΔAla^6^] fusaricidin LI-F07a, the *F. asiaticum* and *B. cinerea* spores were collected. The spore concentration of each pathogen was adjusted to 10^8^ CFU/mL. A total of 1 mL of each spore suspension was added to 20 mL of warm and melted PDA medium. Three aseptic rings made of stainless steel were placed onto the solidified medium plate, and 100 μL of LI-F07a (20 mg/L) and 100 μL of [ΔAla^6^] fusaricidin LI-F07a (20 mg/L) were injected into two separate rings. Then, 100 μL of methanol, which dissolved the above two substances, was injected into the ring of the medium plate as a control. After 4 days of culture at 28°C, the diameter of the inhibition zone could be observed and recorded.

### 2.5. Control effect assays

To test the control effect of fusaricidin LI-F07 and its novel derivative against plant pathogen *in vivo*, we used two pathogen fungi to infect the cucumber seedlings as the model according to a previous method (Li et al., 2015). Initially, the cucumber seedlings (*Cucumis sativus* Linn.) were planted in the mixture containing 150 g peat soil and 50 g vermiculite in 10-cm pots. When the seedlings reached the three-leaves period, 10 mL of fusaricidin LI-F07a (20 mg/L) and its novel derivative (20 mg/L) was sprayed onto the seedling leaves, with 10 mL of water being used as the control. At 12 h after spray, 5 mL of spore suspensions (10^8^ cfu/mL) collected from two pathogen fungi (*F. oxysporum* and *B. cinerea*) was inoculated by foliar spray. Each group contained eight seedlings. At 21 days post-inoculation, we investigated the disease severity by the following grades: 0, number of leaves with no symptoms; 1, number of leaves with <25% area with disease spots; 2, number of leaves with 25–50% area with disease spots; 3, number of leaves with 50–75% area with disease spots; 4, number of leaves with 75–100% area with disease spots; 5, number of leaves with 100% area with disease spots. The disease index (DI) was determined by the formula below (Yan et al., 2006):

$$DI=\frac{\sum \left(g\times N_{g}\right)}{h\times N_{t}}$$

Where *g* is the grade value, *N_g_* is the number of leaves of the corresponding grade, *h* is the highest-grade value, and *N_t_* is the total number of leaves in each group.

The control effect (CE) was calculated as follows (Yan et al., 2006):

$$CE\left(\%\right)=\left[1-\frac{{DI}_{\mathrm{treatment}}}{{DI}_{\mathrm{control}}}\right]\times 100\%$$

### 2.6. Extraction, purification, and identification of antifungal component

To extract the purified fusaricidin or its potential derivative, the wild-type strain or ΔM6-engineered strain was cultivated in 100 mL of KL broth at 37°C for 3 days at 220 rpm. The fermentation was extracted by 10 mL of ethyl acetate for 5 h at 4°C. Then, the ethyl acetate phase was dried. The strain pellets were extracted in methanol by vigorously shaking for 1 h. The weight of methanol was twice the weight of the strain. The methanol supernatant collected by centrifugation was then dried. The two dried components could be dissolved in 1 mL of methanol.

To analyze the difference of the crude extractions between the wild-type strain and ΔM6 mutant, we compared their chromatography peaks via analytical HPLC (Shimadzu LC-20AT) at UV 210 nm with a C18 reversed-phase column (150 × 4.6 mm). The condition for detecting fusaricidin has been described previously with some modifications (Li et al., 2007). It is listed as follows: injection volume 10 μL, flow rate 0.8 mL/min, and solvent gradient ranging from 40% B (acetonitrile) with 60% D (0.1% formic acid in water) to 90% B (acetonitrile) with 10% D (0.1% formic acid in water) in 30 min.

To acquire the antifungal substances, we purified the crude extractions using preparative HPLC (Shimadzu LC-16P) at UV 210 nm by the following method: 1 mL of crude extract was injected into a C18 reversed-phase column (250 × 10 mm) with 0.1% trifluoroacetic acid in 90% acetonitrile solution at 20 mL/min. Each peak was collected specifically for antifungal activity assays, and only the peaks showing activity were selected for subsequent LC–MS analysis.

The conditions for LC–MS (Agilent 1,290-6470A) were conducted under positive mode as described previously, with minor modifications (Vater et al., 2015). The conditions for LC isolation were as follows: 30% A (0.1% formic acid in water) with 70% B (0.1% formic acid in methanol) and flow rate 0.45 mL/min. The conditions for MS were as follows: gas temperature 300°C, drying gas flow 5 L/min, nebulizer 30 psi, capillary voltage 3.5 kV, sheath gas heater 350°C, sheath gas flow 11 L/min, and fragmentation voltage 140 V. When performing MS–MS, an additional parameter was collision voltage (10 V), while all other conditions remained as described above.

### 2.7. Statistical analysis

All the experiments were repeated three times with a similar result. The significant difference (P<0.01) of data was analyzed by one-way ANOVA with Duncan’s multiple-range test using SPSS version 22 statistical software (Chicago, United States).

## 3. Results

### 3.1. *Paenibacillus polymyxa* possesses FusA coding gene and produces fusaricidin LI-F07a

**Figure 1:**
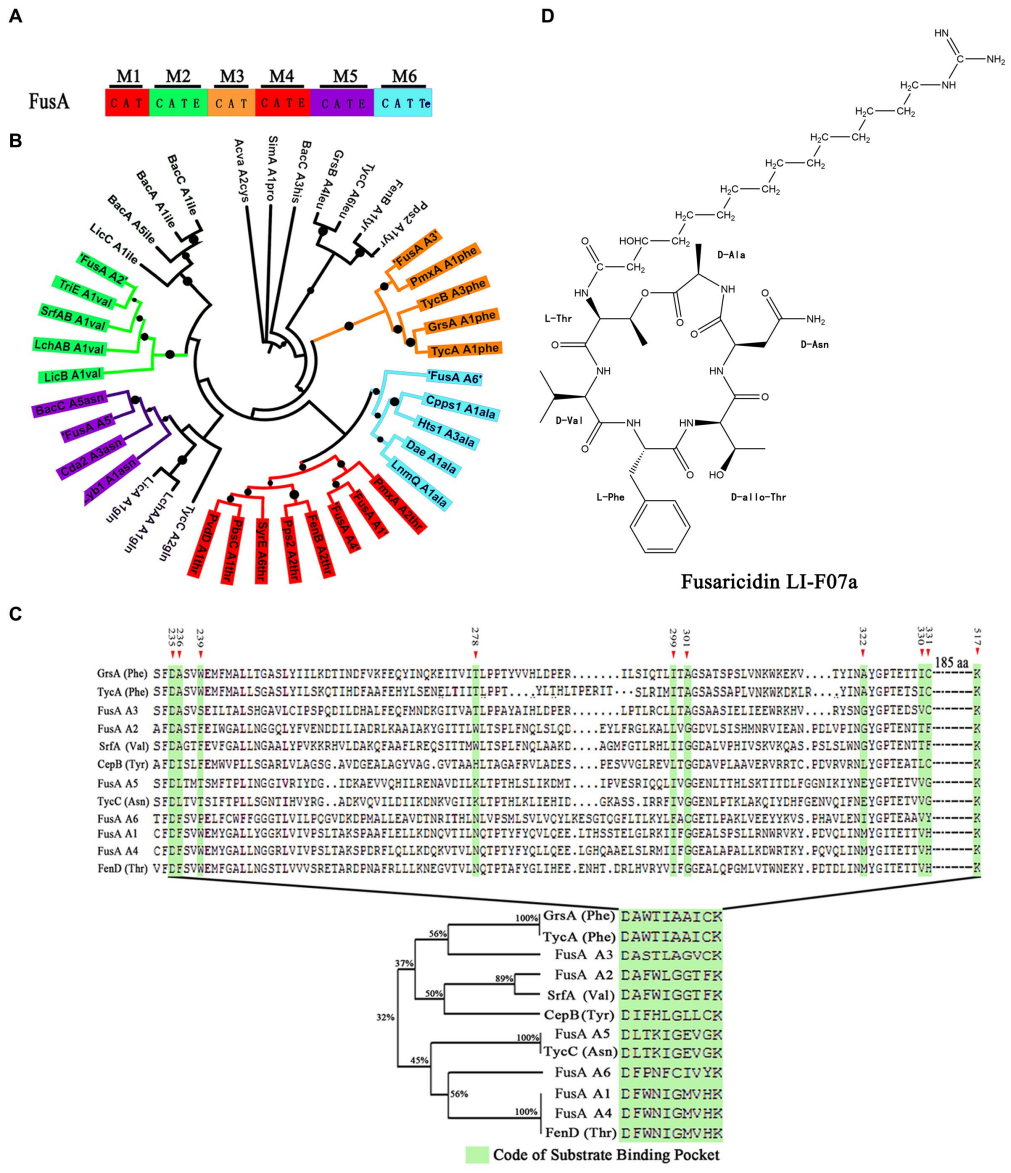
**(A)** Module and domain organization of FusA in *P. polymyxa*. C indicates condensation domain, A indicates adenylation domain, E indicates epimerization domain, T indicates thiolation domain, and TE indicates thioester domain. **(B)** The phylogenetic tree of A domains within FusA and within other NRPSs. The black dot indicates bootstrap value. Thr indicates threonine, val indicates valine, phe indicates phenylalanine, asn indicates asparagine, ala indicates alanine, ile indicates isoleucine, and gln indicates glutamine. **(C)** Alignment based on the ten code residues. The green shadow box indicates the “ten code residues” located at positions 235, 236, 239, 278, 299, 301, 322, 330, 331, and 517 within the A domain, serving as the binding site for the A domain when interacting with the substrate in the three-dimensional structure. **(D)** Structure of fusaricidin LI-F07a.

To predict whether our wild-type strain could produce fusaricidin LI-F07a, we analyzed its genome sequence and found the FusA coding gene. FusA harbors six highly similar modules consisting of at least three domains: an adenylation (A), a thiolation (T), and a condensation (C) domain. In addition, there is an epimerization (E) domain in the second, fourth, and fifth modules and a termination (TE) domain in the sixth module (Figure 1A).

To determine the substrate of each A domain within FusA, we compared each of the A domains within FusA with those known substrate-activating A domains and aligned them. As shown in Figure 1B, phylogenetic analysis revealed the potential substrate specificity of these A domains within each module: the first A domain of FusA (FusA-A1) could recognize threonine (Thr), the second A domain of FusA (FusA-A2) could recognize valine (Val), the third A domain of FusA (FusA-A3) could recognize phenylalanine (Phe), the fourth A domain of FusA (FusA-A4) could recognize threonine (Thr), the fifth A domain of FusA (FusA-A5) could recognize asparagine (Asn), and the sixth A domain of FusA (FusA-A6) could recognize alanine (Ala). A similar sequence alignment based on the “ten code residues” also demonstrated that the six A domains within FusA in strain WLY78 could, respectively, recognize Thr, Val, Phe, Thr, Asn, and Ala (Figure 1C). Therefore, FusA is supposed to biosynthesize hexadepsipeptide in the following order: Thr^1^-Val^2^-Phe^3^-Thr^4^-Asn^5^-Ala^6^, which is a part of fusaricidin LI-F07a (Figure 1D).

**Figure 2:**
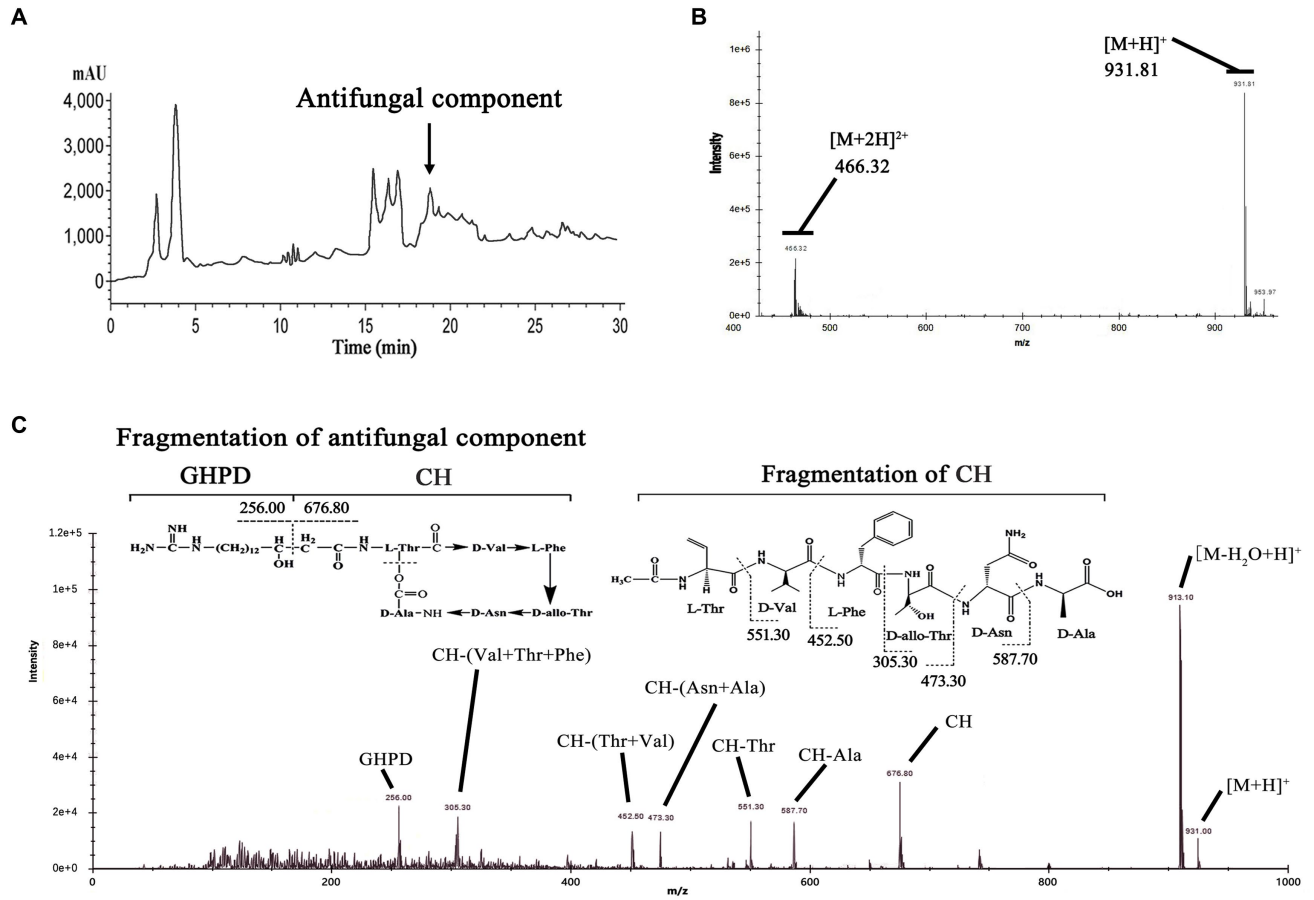
**(A)** Analytical HPLC profile at UV 210 nm of crude extracts from wild-type strain carrying FusA. The active ingredient indicated by the arrow is fusaricidin LI-F07a, which was later detected by activity tests and mass spectrometry. **(B)** Mass spectrum analysis of antifungal component peak. The number 931.81 indicates the mass of fusaricidin LI-F07a. **(C)** MS–MS analysis of peaks at m/z 931.81 as a precursor. CH indicates cyclic hexapeptide. GHPD indicates 15-guanidino-3-hydroxypentadecanoic acid.

To verify our prediction, we prepared *P. polymyxa* strain fermentation and crude extractions. Then, we analyzed the extractions and collected a component peak that showed strong antifungal activity by HPLC at 18.7 min (Figure 2A). This active component was identified by LC–MS and yielded an ion peak at m/z = 931.81, which represents the ratio of the mass of the protonated fusaricidin LI-F07a to its charge (Figure 2B). Subsequently, the ion of m/z of 931.81 was used as precursor for MS–MS fragmentation. As shown in Figure 2C, the ion of m/z 256.07 represents the 15-guanidino-3-hydroxypentadecanoic acid side chain, and the ion of m/z 676.54 represents the cyclic hexadepsipeptide fragment. The N-terminal stepwise cleavage of cyclic hexadepsipeptide was Thr (551.41), Val (452.19), Phe (305.11), and Thr (473.44), while the C-terminal stepwise cleavage of cyclic hexadepsipeptide was Ala (587.65) and Asn (473.44). In all, these fragment ions are completely consistent with the molecular structure of fusaricidin LI-F07a, demonstrating that fusaricidin LI-F07a could be synthesized by *P. polymyxa*.

### 3.2. Modifying the 6th FusA module enhances the antifungal activity of bacterium

**Figure 3:**
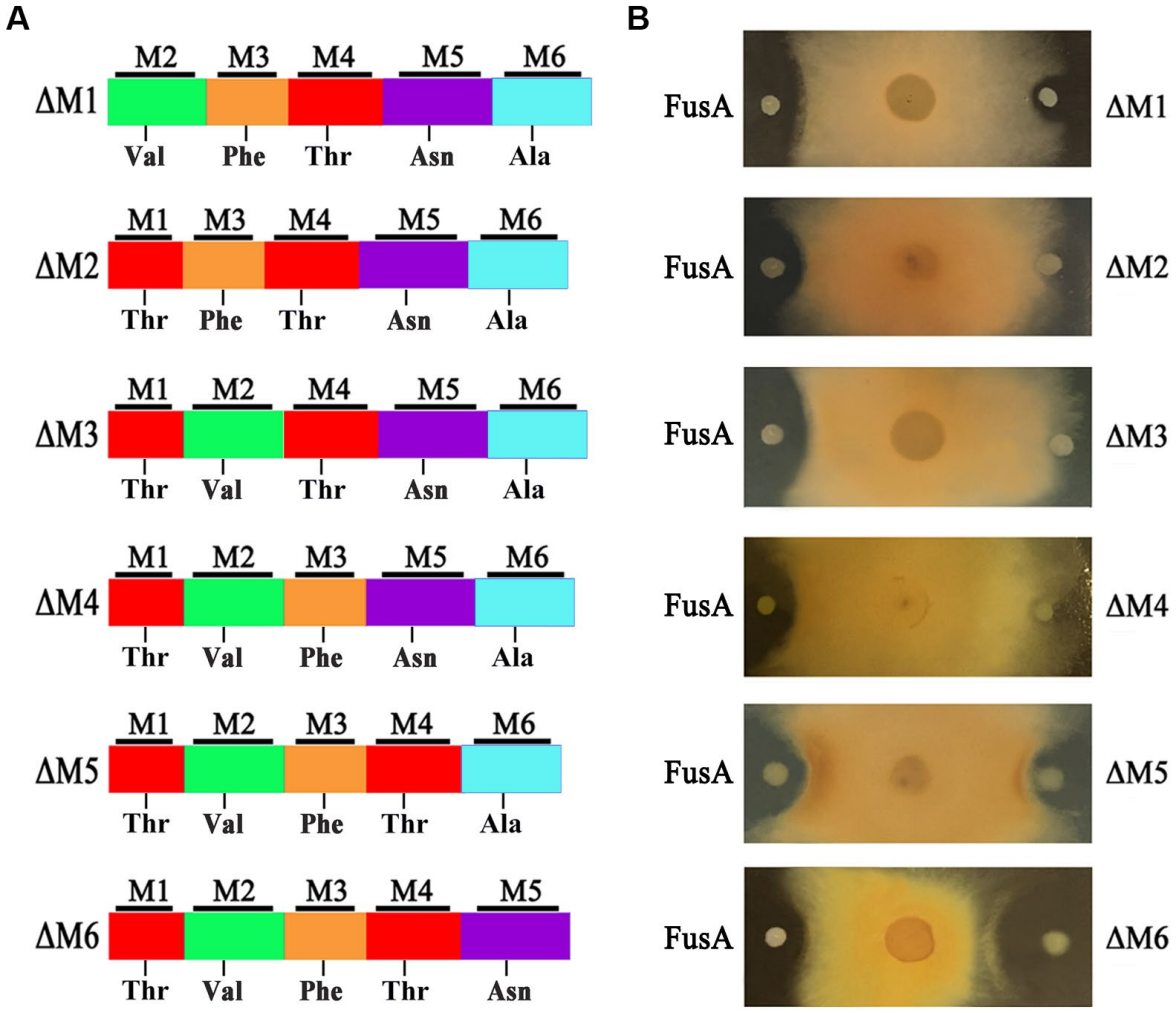
**(A)** Module organization of modified FusA and its substrate. **(B)** The inhibition effect of wild-type strain containing FusA and engineered strain carrying modified FusA against *F. asiaticum*.

To design a new and more efficient antibiotic fusaricidin against *Fusarium* fungi, we inactivated each of the six modules within FusA, yielding six engineered strains that, respectively, contain different types of FusAs: ΔM1, ΔM2, ΔM3, ΔM4, ΔM5, and ΔM6 (Figure 3A). Each engineered strain was confirmed by PCR analysis (Supplementary Figure S2). As a consequence, the inhibiting effect against *F. asiaticum* of two engineered strains with ΔM1 and ΔM5 decreased slightly. Those engineered strains carrying ΔM2, ΔM3, and ΔM4 completely lost their antifungal activities. Meanwhile, the strain with ΔM6 showed a larger inhibition zone than the wild-type strain, suggesting that ΔM6 might produce a more efficient compound rather than fusaricidin LI-F07a (Figure 3B).

### 3.3. The new compound is characterized as [ΔAla^6^] fusaricidin LI-F07a

**Figure 4:**
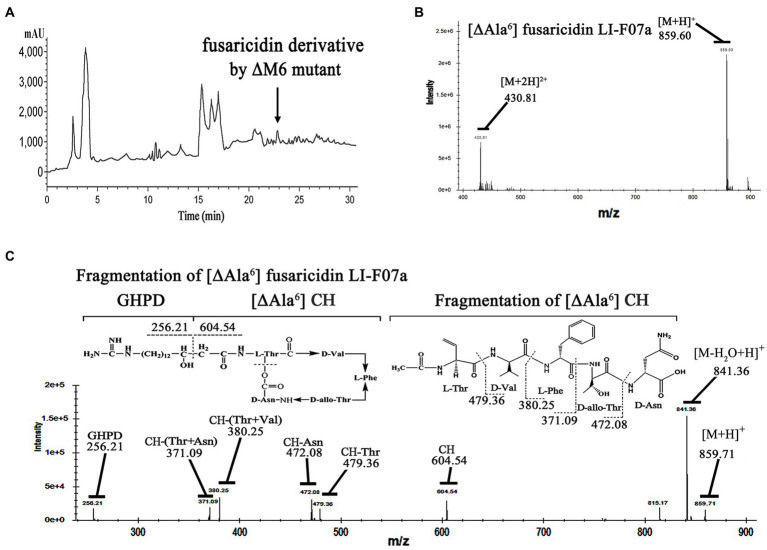
**(A)** Analytical HPLC profile at UV 210 nm of crude extracts from engineered strain carrying ΔM6. The active ingredient indicated by the arrow is [ΔAla^6^] fusaricidin LI-F07a, which was later detected by activity tests and mass spectrometry. **(B)** Mass spectrum analysis of antifungal component peak. The number 859.60 indicates the mass of [ΔAla^6^] fusaricidin LI-F07a. **(C)** MS–MS analysis of peaks at m/z 856 as a precursor. CH indicates cyclic hexapeptide. GHPD indicates 15-guanidino-3-hydroxypentadecanoic acid.

To identify the structure of this compound, we isolated and detected this active component from the fermentation broth of the ΔM6-engineered strain by HPLC at 22.8 min (Figure 4A). Then, we analyzed this fraction by LC–MS and observed an ion peak at 859.60 Da (Figure 4B), which was ~72 Da (the mass of Ala) smaller than fusaricidin LI-F07 (931.81 Da), suggesting this compound lacks the sixth amino acid (Ala) from fusaricidin LI-F07a. To further confirm its structure, we used the ion 859.60 Da as a precursor for MS–MS fragment ion analysis. As shown in Figure 4C, the fragment ion 256.21 Da represents the 15-guanidino-3-hydroxypentadecanoic acid side chain, and the ion 604.54 Da represents the cyclic hexadepsipeptide of this new substance. The N-terminal stepwise cleavage of cyclic hexadepsipeptide was Thr (479.36 Da) and Val (380.25 Da), while the C-terminal stepwise cleavage of cyclic hexadepsipeptide was Asn (472.08 Da) and Thr (371.09 Da). Therefore, we believe that the fracture fragments from ion 859.71 Da roughly match the molecular fragmentation pattern of [ΔAla^6^] fusaricidin LI-F07a, which was identified as a novel fusaricidin derivative.

### 3.4. [ΔAla^6^] Fusaricidin LI-F07a exhibits great antimicrobial ability both *in vitro* and *in vivo*

**Figure 5:**
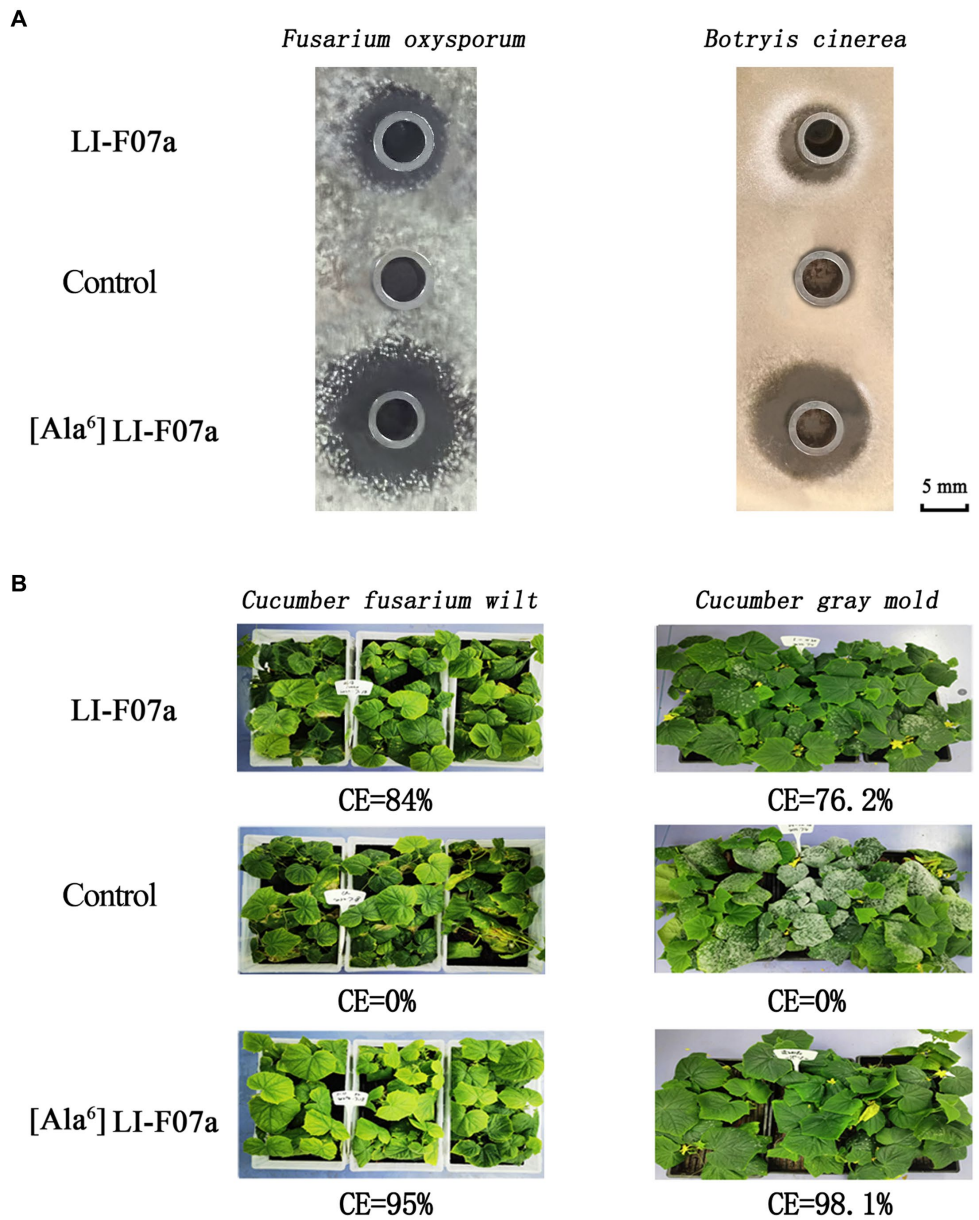
**(A)** Antifungal activity of fusaricidin LI-F07a and [ΔAla^6^] fusaricidin LI-F07a against *F. oxysporum* and *B. cinerea*. **(B)** Control effect of fusaricidin LI-F07a and [ΔAla^6^] fusaricidin LI-F07a against cucumber *fusarium* wilt and cucumber gray mold.

With the same concentration at 20 mg/L, [ΔAla^6^] fusaricidin LI-F07a shows 1-fold higher antifungal activities against *F. oxysporum* and *B. cinerea* than that of fusaricidin LI-F07a (Figure 5A). Meanwhile, the CE values of [ΔAla^6^] fusaricidin LI-F07a against cucumber *fusarium* wilt and cucumber gray mold reach 95 and 98.1%, while those of fusaricidin LI-F07a only reach 84 and 76.2%, respectively (Figure 5B).

## 4. Discussion

The *Paenibacillus* species is a powerful bio-fungicide and has many potential applications. So far, 14 fusaricidin analog members have been reported to be produced by *P. polymyxa* (Kuroda et al., 2001; Choi et al., 2008). In the present study, by synthetase engineering, we created a biosynthetic pathway for the production of a novel fusaricidin derivative, which could serve as an antibiotic replacement solution for preventing pathogen fungi infection in agriculture.

In the past, to elucidate the structure of a compound produced by a microorganism required fermentation, purification, and then identification. Now, the genome mining method has rapidly paved the way for predicting secondary metabolites within microorganisms. Similarly, we used the genome mining strategy to predict the genome and found the FusA coding gene, suggesting that FusA producing fusaricidin LI-F07a is a great possibility (Figure 1A). The A domain within NRPS is reported to be essential in recognizing the substrate that consists of lipopeptide in a 1:1 manner (Marahiel et al., 1997; Stachelhaus et al., 1999). According to the principle above, a lot of known A domains were aligned with our A domains within FusA, and then, we acquired the substrate of our A domains, which matches the structure of peptide moiety within fusaricidin LI-F07a (Figure 1B). Furthermore, we also noticed the “ten code residues” located at positions 235, 236, 239, 278, 299, 301, 322, 330, 331, and 517 within the A domain. It has been previously reported that these residues serve as the binding site for the A domain when interacting with the substrate in the three-dimensional structure (Conti et al., 1997; Stachelhaus et al., 1999). Since the “ten code residues” are extracted from the A domains, the alignment of either the “A domain” or the “ten code residues” supports the same conclusion (Figure 1C).

Mass spectrometry fragment ion analysis is a reliable detection method and particularly powerful for the structural characterization of analogous compounds (Kurusu et al., 1987; Han et al., 2012). This method has successfully characterized more than 10 fusaricidin members (Vater et al., 2015). With a similar method, we demonstrated that our strain could produce fusaricidin LI-F07a by LC–MS–MS fragment ion analysis (Figure 2). All of the fusaricidin members are lipid-modified non-ribosomal cyclic hexadepsipeptides containing four D-amino acids and two L-amino acids. This particular ω-functionalized lipid side chain is of key importance for the antibiotic activity of the fusaricidins and their selective inhibition of fungal cells due to the interaction with phospholipid cell membranes (Bionda et al., 2012). However, these fusaricidin members with different activity share the same lipid side chain except the cyclic hexadepsipeptide (Kurusu et al., 1987). How much the cyclic hexadepsipeptide of fusaricidin contributes to its antifungal activity still needs to be explored. Therefore, we intend to modify the cyclic hexadepsipeptide of fusaricidin LI-F07a to see whether its antifungal activity is affected.

In past decades, a rational design of novel peptide antibiotics method has been developed by modifying one domain and domain fusion within NRPS in *Bacillus* sp. *and Paenibacillus* sp. (Stachelhaus et al., 1995; Mootz et al., 2000; Yakimov et al., 2000; Han et al., 2012). However, only modifying one domain may cause a decrease in substrate-recognition ability due to the incompatibility of other domains in the same module. This is because, in addition to the A domain in NRPS, other domains such as C, T, and E also exhibit specific substrate recognition. If we could knock out an entire module from the NRPS without causing frameshift mutations, some instances might result in the production of a peptide that is missing a corresponding amino acid.

Therefore, we did not replace any A domains but directly deleted each of modules that consist of the A domain and its neighboring domains (Figure 3A). Excluding the pleiotropic effect of a mutation in prokaryotes is generally considered easier compared to eukaryotic cells. In prokaryotes, such as our strain, the genome is typically continuous without introns. Introns are non-coding regions in the genome of eukaryotes that can contribute to the pleiotropic effects observed during gene knockout by introducing complexity in gene expression regulation and splicing. However, in prokaryotes, the absence of introns means that gene knockout does not involve the splicing or regulatory complexities associated with introns (Setubal et al., 2018). Moreover, frameshift mutations are one of the main factors that can contribute to the pleiotropic effects of gene knockout. When designing primers for homologous recombination double exchange, we have meticulously considered and designed the primers to effectively prevent any subsequent frameshift mutations resulting from the knockout. Even if the subsequent primary amino acid sequence changes due to a frameshift mutation, a frameshift protein may still retain its structure and functionality. In fact, deleterious frameshift mutations have been proposed to be potential drivers for molecular evolution. Based on this shiftability, many genes and certain genomes are naturally optimized for frameshift tolerance (Wang et al., 2022). Importantly, we ensured the absence of frameshift mutations by conducting thorough sequencing analysis. We successfully acquired a strain with engineered ΔM6 that showed a more enhanced antifungal activity than the wild-type strain with FusA (Figure 3B). However, a former study had created a mutant with inactivated 4′-phosphopantetheinyl transferase, which could disrupt the production of NRPS/NPKS-dependent metabolites such as fusaricidins and still kept its antagonistic activity against *Fusarium culmorum*. They emphasized that the biofilm matrix formation may be of major importance in its antagonism (Timmusk et al., 2019). There could potentially exist an antagonistic relationship between fusaricidins and exopolysaccharides production. It has been demonstrated that fusaricidin inhibits *Bacillus subtilis* by specifically targeting the mechanism of exopolysaccharide production (Yu et al., 2012). Additionally, we observed a similar phenomenon where the capacity for biofilm synthesis was enhanced in ΔM2, ΔM5, and ΔM6 mutants. This enhancement may be associated with increased polysaccharides production. However, except for ΔM6, none of these strains produced antibacterial effects in our study. Therefore, we suppose that the enhanced effect of this engineered strain should be attributed to the new compound [ΔAla^6^] fusaricidin LI-F07a (Figure 4C). Indeed, both *in vitro* and *in vivo*, [ΔAla^6^] fusaricidin LI-F07a did show stronger ability against *F. oxysporum* and *B. cinerea* than fusaricidin LI-F07a (Figure 5).

Until the completion of our study, it remained inconclusive whether the fungicidal activity of our mutants was affected by small peptides or the failure of peptide chains to cyclize due to the early peptide chain breakage caused by knocking out. Usually, the production of fusaricidin LI-F07a ranges from 12 mg/L to 76 mg/L (Raza et al., 2010; Han et al., 2012). In our wild-type strain, the maximum yield of fusaricidin LI-F07a is ~60 mg/L. In the engineered strain M6, the production of [ΔAla^6^] fusaricidin LI-F07a is ~55 mg/L. Based on the comparison of the yield data before and after the genetic modification, it can be observed that the synthesis efficiency of the derivative is reduced by approximately 10 percent. The engineered ΔM6 shows promising potential as a sustainable choice that can meet diverse industrial and agricultural demands. [ΔAla^6^] fusaricidin LI-F07a can be applied effectively to prevent plants from being infected by *Fusarium* species pathogens.

## Data availability statement

The original contributions presented in the study are included in the article/Supplementary material, further inquiries can be directed to the corresponding authors.

## Author contributions

YL performed the research and drafted the manuscript. SC made crucial revisions of the manuscript. All authors contributed to the article and approved the submitted version.

## Funding

The funders of this work are Chengdu NewSun Crop Science Company Limited (No. SWHCRR001) and the National Key Research and Development Program of China Award (No. 2017YFD0200807).

## Conflict of interest

YL was employed by Chengdu NewSun Crop Science Co. Ltd.

The authors declare that this study received funding from Chengdu NewSun Crop Science Co. Ltd. This funder was engaged in the purifications, the greenhouse trails and the writing of this article.

## Publisher’s note

All claims expressed in this article are solely those of the authors and do not necessarily represent those of their affiliated organizations, or those of the publisher, the editors and the reviewers. Any product that may be evaluated in this article, or claim that may be made by its manufacturer, is not guaranteed or endorsed by the publisher.
