# Supplementary material for: Structure modification of an antibiotic: by engineering the fusaricidin bio-synthetase A in Paenibacillus polymyxa
Source: Front Microbiol. 2023 Sep 26;14:1239958. doi: 10.3389/fmicb.2023.1239958 (PMC10562733; doi:10.3389/fmicb.2023.1239958)
Supplement: Supplementary file 1 [file Data_Sheet_1.docx]

*Supplementary Materials*

Structure modification of an antibiotic: by engineering the fusaricidin bio-synthetase A in *Paenibacillus polymyxa*

**Yunlong Li^1,2^* and Sanfeng Chen^2^*.**

^1^ Chengdu NewSun Crop Science Co., Ltd, No. 35 Gongye south Road, Chengdu, 611630, PR. China.

^2^ State key laboratory of Agrobiotechnology, College of Biological Sciences, China Agricultural University, No. 2 Yuanmingyuan West Road, Beijing 100193, PR. China.

* Corresponding author: chensf@cau.edu.cn; Tel: +86-10-62732674.

**Supplementary Tables**

**Table** **S1.** Strains and plasmids

| **Strains and plasmids** | **Description** | **Source** |
| --- | --- | --- |
| **Bacterial strains** |  |  |
| *Escherichia coli* DH5α | *sup*E44Δ*lac*U169 (φ80 *lacZ*ΔM15) *hsd*R17 *recA1 end* *A1* *gyrA96* *thi-1* *relA1* | TaKaRa |
| ***Paenibacillus polymyxa*** |  |  |
| WLY78 | Wild-type | This work |
| ΔM1 mutant | In-frame deletion of 1^st^ module from FusA | This work |
| ΔM2 mutant | In-frame deletion of 2^nd^ module from FusA | This work |
| ΔM3 mutant | In-frame deletion of 3^rd^ module from FusA | This work |
| ΔM4 mutant | In-frame deletion of 4^th^ module from FusA | This work |
| ΔM5 mutant | In-frame deletion of 5^th^ module from FusA | This work |
| ΔM6 mutant | In-frame deletion of 6^th^ module from FusA | This work |
| **Fungi** |  |  |
| *Fusarium* *asiaticum* | Phytopathogen fungi | ACCC |
| *Fusarium oxysporum* | Phytopathogen fungi | ACCC |
| *Botrytis cinerea* | Phytopathogen fungi | ACCC |
| **Plasmids** |  |  |
| pRN5101 | Temperature-sensitive shuttle vector, *Erm^R^* | TaKaRa |
| pRN-M1 | A pRN5101 derivative carrying upstream and downstream fragments of M1 for constructing M1 deletion mutant | This work |
| pRN-M2 | A pRN5101 derivative carrying upstream and downstream fragments of M2 for constructing M2 deletion mutant | This work |
| pRN-M3 | A pRN5101 derivative carrying upstream and downstream fragments of M3 for constructing M3 deletion mutant | This work |
| pRN-M4 | A pRN5101 derivative carrying upstream and downstream fragments of M4 for constructing M4 deletion mutant | This work |
| pRN-M5 | A pRN5101 derivative carrying upstream and downstream fragments of M5 for constructing M5 deletion mutant | This work |
| pRN-M6 | A pRN5101 derivative carrying upstream and downstream fragments of M6 for constructing M6 deletion mutant | This work |

**Table** **S2.** Synthtase and the GenBank No.

| **Synthetase** | **Accession No.** | **Synthetase** | **GenBank No.** |
| --- | --- | --- | --- |
| Acva | CAA38631.1 | LicC | AAD04759.1 |
| BacA | AAC06346.1 | LnmQ | AF484556.1 |
| BacC | AAC06348.1 | Lyb1 | CAA65394.1 |
| Cda2 | CAB38517.1 | PbsC | CAA54778.1 |
| Cpps1 | CAB39315.1 | PmxA | AEZ51516.1 |
| Dae | AAA25234.1 | Pps2 | CAA84361.1 |
| FenB | AAB00093.1 | PvdD | AAB60198.1 |
| GrsA | CAA33603.1 | SimA | CAA82227.1 |
| GrsB | CAA43838.1 | SrfAB | BAA08983.1 |
| Hts1 | M98024.2 | SyrE | AAC80285.1 |
| LchAA | CAA06323.1 | TriE | AKH45460.1 |
| LchAB | CAA06324.1 | TycA | AAC45928.1 |
| LicA | AAD04757.1 | TycB | AAC45929.1 |
| LicB | AAD04758.1 | TycC | AAC45930.1 |

**Table** **S3.** Primers for modification of FusA

| **PCR product** | **Primer** | **Oligonucleotide sequences (5’-3’)** |
| --- | --- | --- |
| Upstream of M1 | M1UF | acgatgcgtccggcgtagagATACAAGCGCCGTCTGTTC |
|  | M1UR | ttctgacggaGTTTCTATTCAAATATTGATAGAGGAACTTAC |
| Downstream of M1 | M1DF | gaatagaaacTCCGTCAGAAATAATATGG |
|  | M1DR | gcgaccacacccgtcctgtgATTAGCAATTCATTTGGTTTC |
| Upstream of M2 | M2UF | acgatgcgtccggcgtagagGCAGTCGCGTCTGGCGTT |
|  | M2UR | ttcagatgggTGGTGTGTTCCGTTAATTACGCAG |
| Downstream of M2 | M2DF | gaacacaccaCCCATCTGAAATGATGTGATGCATATCAAAC |
|  | M2DR | gcgaccacacccgtcctgtgTGGGGCGGATCGACCATC |
| Upstream of M3 | M3UF | acgatgcgtccggcgtagagCCACTGCATTCGGAGTAC |
|  | M3UR | catggatgggGTTTCTATGAATATTCTCATAGAAGAG |
| Downstream of M3 | M3DF | tcatagaaacCCCATCCATGACGATGTG |
|  | M3DR | gcgaccacacccgtcctgtgTATACCGAATATTGACATCACC |
| Upstream of M4 | M4UF | acgatgcgtccggcgtagagTTATCCGGCGTACGGGCC |
|  | M4UR | ctcagatgggTGGTGTATCCCGATCATTACACAG |
| Downstream of M4 | M4DF | ggatacaccaCCCATCTGAGACAATATG |
|  | M4DR | gcgaccacacccgtcctgtgAGTACTTAGGTCGAATAGATC |
| Upstream of M5 | M5UF | acgatgcgtccggcgtagagTCCGGTGTGAGCGCAGCT |
|  | M5UR | tatggatggcTGGTGTATGCCACTTGTGCTCC |
| Downstream of M5 | M5DF | gcatacaccaGCCATCCATAACAATATGG |
|  | M5DR | gcgaccacacccgtcctgtgAAATGATCATACCAGATCTG |
| Upstream of M6 | M6UF | acgatgcgtccggcgtagagGAGCATATGATCATCCATGATCAAC |
|  | M6UR | cggtagtattTTCAAGCTGCAGGCAACAAC |
| Downstream of M6 | M6DF | gcagcttgaaAATACTACCGATATGCCGTG |
|  | M6DR | gcgaccacacccgtcctgtgTGTGGATGGCGTATCCTG |

**Supplementary Figures**

**
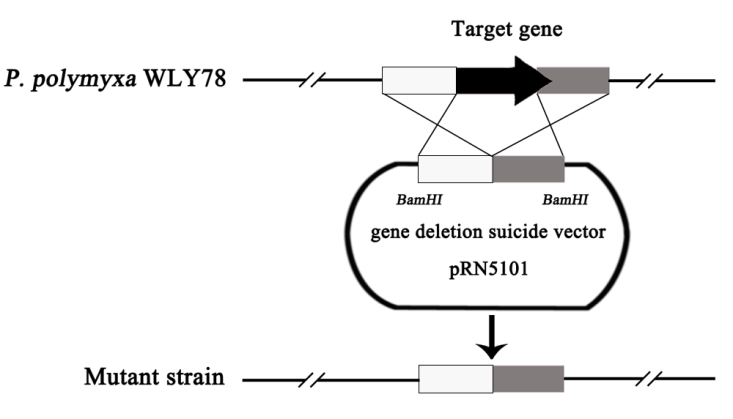
**

**Figure S1.** **General scheme for construction of deletion mutants.** Two DNA fragments upstream (white) and downstream (grey) the target gene (black) were PCR amplified from the genome DNA of *P. polymyxa* WLY78 and then were assembled to suicide vector pRN5101 digested with *Bam*HΙ. Mutants ΔM1, ΔM2, ΔM3, ΔM4, ΔM5 and ΔM6 were constructed in this way.


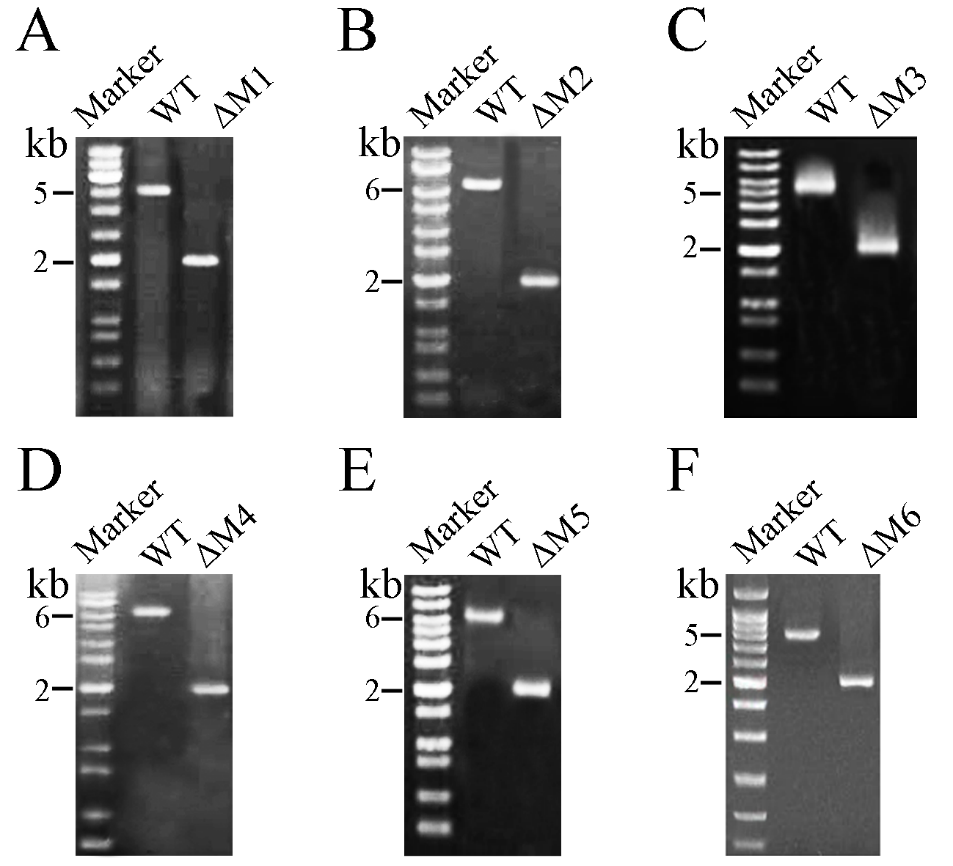


**Figure S2. Agarose gel electrophoresis of ΔM1, ΔM2, ΔM3, ΔM4, ΔM5, ΔM6.** WT and ΔM1 indicate PCR fragments amplified by primers UM1F/DM1R respectively using WLY78 DNA and ΔM1 DNA as template indicates. The amplicon from ΔM1 mutant was 3.1 kb smaller than that of WT (**A**). WT and ΔM2 indicate PCR fragments amplified by primers UM2F/DM2R respectively using WLY78 DNA and ΔM2 DNA as template indicates. The amplicon from ΔM2 mutant was 4.5 kb smaller than that of WT (**B**). WT and ΔM3 indicate PCR fragments amplified by primers UM3F/DM3R respectively using WLY78 DNA and ΔM3 DNA as template indicates. The amplicon from ΔM3 mutant was 3 kb smaller than that of WT (**C**). WT and ΔM4 indicate PCR fragments amplified by primers UM4F/DM4R respectively using WLY78 DNA and ΔM4 DNA as template indicates. The amplicon from ΔM4 mutant was 4.5 kb smaller than that of WT (**D**). WT and ΔM5 indicate PCR fragments amplified by primers UM5F/DM5R respectively using WLY78 DNA and ΔM5 DNA as template indicates. The amplicon from ΔM5 mutant was 4.5 kb smaller than that of WT (**E**). WT and ΔM6 indicate PCR fragments amplified by primers UM6F/DM6R respectively using WLY78 DNA and ΔM6 DNA as template indicates. The amplicon from ΔM6 mutant was 3.1 kb smaller than that of WT (**F**).
